# Are there sex differences in cardiovascular outcomes in non-dialysis CKD patients?

**DOI:** 10.1093/ckj/sfad174

**Published:** 2023-08-16

**Authors:** Pasquale Fabio Provenzano, Grazia Caridi, Giovanna Parlongo, Daniela Leonardis, Elvira Puntorieri, Giovanni Tripepi, Carmine Zoccali, Francesca Mallamaci

**Affiliations:** CNR-IFC, Clinical Epidemiology and Physiopathology of Renal Diseases and Hypertension, Reggio Calabria, Italy; Nephrology, Dialysis, and Transplantation Unit, Grande Ospedale Metropolitano (GOM), Reggio Calabria, Italy; Nephrology, Dialysis, and Transplantation Unit, Grande Ospedale Metropolitano (GOM), Reggio Calabria, Italy; CNR-IFC, Clinical Epidemiology and Physiopathology of Renal Diseases and Hypertension, Reggio Calabria, Italy; Nephrology, Dialysis, and Transplantation Unit, Grande Ospedale Metropolitano (GOM), Reggio Calabria, Italy; CNR-IFC, Clinical Epidemiology and Physiopathology of Renal Diseases and Hypertension, Reggio Calabria, Italy; Renal Research Institute NY, USA; BIOGEM, Ariano Irpino; IPNET, Reggio Calabria, Italy; CNR-IFC, Clinical Epidemiology and Physiopathology of Renal Diseases and Hypertension, Reggio Calabria, Italy; Nephrology, Dialysis, and Transplantation Unit, Grande Ospedale Metropolitano (GOM), Reggio Calabria, Italy

**Keywords:** cardiovascular disease, CKD, female, risk assessment, risk difference

## Abstract

**Background:**

Sex differences for cardiovascular (CV) risk and outcomes in chronic kidney disease (CKD) patients not on dialysis have been scarcely or never investigated. We therefore studied this important aspect in a cohort of CKD stage 2–5 in the south of Italy.

**Methods:**

We tested the relationship between sex and fatal and non-fatal major CV events in a cohort of 759 stage 2–5 CKD patients followed up for a median time of 36 months.

**Results:**

Out of 759 patients, 455 were males (60%) and the remaining 304 patients were females (40%). During the follow-up, 42 patients died, and 118 had fatal and non-fatal CV events. On univariate Cox regression analyses, the male sex failed to be associated with all-cause mortality but was strongly related to the incidence rate of fatal and non-fatal major CV events [hazard ratio (HR) 1.75, 95% confidence interval (CI) 1.18–2.60, *P* = .006]. Data adjustment for a series of major potential confounders did not materially affect the strength of this relationship (HR 1.78, 95% CI 1.03–3.09). Further analysis testing the effect of age on major CV outcomes by sex showed an effect modification by this risk factor on the same outcome (*P* = .037) because the HR of male versus female CV events increased progressively with aging.

**Conclusion:**

Male patients in stage G2–5 CKD had a higher risk for CV events compared with female patients. Age was shown to be a risk modifier for the association between sex and CV events and this risk increased linearly across a wide age spectrum in CKD patients.

## INTRODUCTION

Female sex discrepancy and weakness is a ‘fact’ in anthropological studies that is hard to deny. However, in other fields of science, such as medicine and risk factors, women always have a predominant and stronger position than men.

Indeed, in every human race and ethnicity, the female sex has a risk for death and many adverse events which is lower than in men. In fact, in Afro-Americans, Caucasians, Asians and Hispanics the risk of death is almost half in women in comparison with men of the same race and ethnicity [1, 2].

While the Framingham cardiovascular (CV) risk factors explain 75% of CV diseases, atherosclerosis and chronic kidney disease (CKD), sex has been documented to be one of the most important CV risk factors together with age. Among medical specialties, the impact of sex in medicine is well documented in heart disease and particularly in coronary heart disease (CHD), which is a rapidly increasing condition in Western countries where it represents one of the most common causes of morbidity and mortality [3]. In a study in the general population, in the USA, CHD was demonstrated to be more prevalent in men than in women throughout all age strata [4].

Moreover, cardiologists are well aware of this phenomenon which has resulted in a number of medical innovations, such as the adaptation of coronary angiography diagnostics to the different pathophysiology of myocardial ischaemia separately in men and women, as well as variations in emergency prioritization listing based on sex-specific symptoms of myocardial infarction [5, 6]. In other specialties such as nephrology, sex is a scarcely explored field of research as far as CV outcomes are concerned in this high-risk population. There is evidence that the progression of kidney disease is affected by sex. Indeed, the biggest meta-analysis to date, including more than 11 000 patients from 68 different studies, demonstrated that renal disease from various causes in women (i.e. polycystic kidney disease, immunoglobulin A nephropathy, membranous glomerulopathy and chronic renal disease of unknown aetiology) progresses at a lower rate than it does in blood pressure– and lipid levels–matched men with these diseases [7, 8]. On the contrary, little or no information is known about CV outcomes and sex differences in the non-dialysis CKD population. The aim of this study was to explore differences in CV outcomes between men and women in a cohort of the non-dialysis CKD population in southern Italy.

## MATERIALS AND METHODS

### Study protocol

The study protocol conformed to the ethical guidelines of our institution and received approval from the ethical committees of all the participating units. Each participant provided written informed consent to take part in the study.

### Patients with CKD

We included in this analysis 759 patients with stage 2–5 CKD (age 62 ± 11 years; 60% male) consecutively recruited from nephrology units in southern Italy (the regions of Calabria, Sardinia and Sicily) who participated in the Multiple Intervention and Audit in Renal Diseases to Optimize Care (MAURO) study. The MAURO study was a cluster randomized controlled trial in 22 renal clinics that aimed to assess the efficacy of a multimodal quality improvement intervention to increase compliance with guideline recommendations for the prevention of CKD progression and CV complications in a CKD population. Patient enrolment was performed between October 2005 and 2008. The selection criteria and the detailed clinical characteristics of this cohort are described elsewhere [9]. The study included six visits over a 3-year follow-up. At enrolment, all patients were in stable clinical condition and none had intercurrent infections or acute inflammatory processes. Inclusion criteria were non-acute or rapidly evolving renal diseases, age ranging from 18 to 75 years, non-transplanted and non-pregnant, and not affected by cancer or diseases in the terminal phase.

### Follow-up and study outcome

After the initial assessment, patients were followed up for a median time of 36 months (range 0.3–48 months). All-cause death and fatal and non-fatal CV events were accurately recorded across time. These events included myocardial infarction, documented by electrocardiography and biomarkers of myocardial injury; heart failure, defined as dyspnoea in addition to two of the following conditions: raised jugular pressure, bi-basilar crackles, pulmonary venous hypertension or interstitial oedema on chest radiography requiring hospitalization; electrocardiography-documented arrhythmia; stroke; peripheral vascular disease; and major arterial or venous thrombotic episodes. These events were accurately recorded during the follow-up period. The history of CVD was defined as the presence of at least one of the following comorbidities at enrolment: myocardial infarction, heart failure, peripheral vascular disease, stroke, transient ischaemic attack or coronary surgery/angioplasty.

### Laboratory measurements

In the whole study population, blood sampling was performed in the early morning after an overnight fast, and plasma was stored at –80°C until analysis. Serum glucose, lipids, haemoglobin, albumin, creatinine and C-reactive protein (CRP) were measured by standard methods in the routine clinical laboratory. The estimated glomerular filtration rate (eGFR) was calculated by using the four-variables Modification of Diet in Renal Disease study equation (MDRD_186_). All CKD patients underwent a 24-h urinary collection for the measurement of urinary protein.

### Statistical analyses

Continuous and categorical variables were expressed as mean ± standard deviation or median and interquartile range (IQR), and as number of participants and percentage, respectively. Survival analysis according to sex was carried out by Kaplan–Meier analysis and by univariate and multiple Cox regression models including a series of potential confounders. The tested covariate included all variables listed in Table 1. In the multiple Cox regression model, we included age and sex as well as variables that significantly differed (*P* < .05) between males and females. To investigate the effect of modification by age on the relationship between sex and fatal and non-fatal CV events we included in the same multiple Cox regression model age (in years), sex (males versus females) and their interaction (multiplicative) terms. As a sensitivity analysis, Cox regression models including the allocation arm were also fitted. The hazard ratios (HRs) of death across age values were calculated by the standard linear combination method. In Cox models data were expressed as HRs, 95% confidence interval (CI) and *P*-value. In Cox models, the proportionality assumption was tested by the analysis of Schoenfeld residuals and no violation was found. All calculations were made by using a standard statistical package (IBM SPSS Statistics for Windows, version 26.0.0., IBM, Armonk, NY, USA).

**Table 1:** Main demographic and clinical characteristics of the whole study population and separately in males and females.

	Whole group	Males	Females	
	(*n* = 759)	(*n* = 455)	(*n* = 304)	*P*-value
Age (years), mean ± SD	62 ± 11	62 ± 11	61 ± 11	.764
Age categories, *n* (%)				
≤25 years	6 (0.8)	3 (0.7)	3 (1.0)	.94
26–35 years	20 (2.6)	12 (2.6)	8 (2.6)	
36–45 years	45 (5.9)	24 (5.3)	21 (6.9)	
46–55 years	98 (12.9)	62 (13.6)	36 (11.8)	
56–65 years	246 (32.4)	149 (32.7)	97 (31.9)	
66–75 years	338 (44.5)	202 (44.4)	136 (44.7)	
≥76 years	6 (0.8)	3 (0.7)	3 (1.0)	
Smokers, *n* (%)	377 (49.7)	325 (71.4)	52 (17.4)	<.001
Diabetes, *n* (%)	265 (34.9)	175 (38.5)	90 (29.6)	.012
With CV comorbidities, *n* (%)	222 (29.2)	162 (35.6)	60 (19.7)	<.001
On oral hypoglycemic therapy, *n* (%)	117 (15.4)	80 (17.6)	37 (12.2)	.129
On insulin therapy, *n* (%)	120 (16.7)	71 (16.5)	49 (17.1)	.833
On erythropoietin therapy, *n* (%)	17 (2.4)	8 (1.9)	9 (3.1)	.269
On diabetes treatment, *n* (%)	214 (29.8)	136 (31.6)	78 (27.2)	.209
On anti-hypertensive treatment, *n* (%)	695 (96.8)	418 (97)	277 (96.5)	.727
On calcium channel blocker therapy, *n* (%)	304 (42.3)	193 (44.8)	111 (38.7)	.105
On ACE inhibitor therapy, *n* (%)	468 (65.2)	293 (68)	175 (61)	.054
On sartan therapy, *n* (%)	299 (39.4)	181(42)	118 (41.1)	.815
On diuretic therapy, *n* (%)	378 (52.6)	229 (53.1)	149 (51.9)	.749
On clonidine therapy, *n* (%)	272 (37.9)	173 (40.1)	99 (34.5)	.127
On statin therapy, *n* (%)	379 (52.8)	225 (52.2)	154 (53.7)	.702
Waist circumference (cm), mean ± SD	99 ± 13	100.9 ± 12.4	96 ± 14.1	<.001
Systolic BP (mmHg), mean ± SD	134 ± 18	133 ± 18	134 ± 18	.631
Diastolic BP (mmHg) , mean ± SD	78 ± 11	77 ± 10	78 ± 111	.678
Heart rate (beats/min), mean ± SD	72 ± 7	72 ± 10	72 ± 10	.476
Creatinine (mg/dL), mean ± SD	2 ± 0.8	2.2 ± 0.8	1.9 ± 0.7	<.001
eGFR_MDRD186_ (mL/min/1.73 m^2^), mean ± SD	35.7 ± 13.3	37.5 ± 13.4	33 ± 12.7	<.001
CKD stages, *n* (%)				
60–89 mL/min/1.73 m^2^	25 (3.3)	18 (4.0)	7 (2.3)	<.001
45–59 mL/min/1.73 m^2^	166 (21.9)	122 (26.8)	44 (14.5)	
30–44 mL/min/1.73 m^2^	292 (38.5)	167 (36.7)	125 (41.1)	
15–29 mL/min/1.73 m^2^	254 (33.5)	144 (31.6)	110 (36.2)	
<15 mL/min/1.73 m^2^	22 (2.9)	4 (0.9)	18 (5.9)	
Urinary protein (g/24 h)	0.6 (0.2–1.5)	0.7 (0.2–1.6)	0.5 (0.2–1.2)	<.001
Glucose (mg/dL), median (IQR)	98 (88–122)	100 (89–122)	97 (86–126)	.138
Cholesterol (mg/dL), mean ± SD	187 ± 45	178 ± 42	199 ± 46	<.001
LDL cholesterol (mg/dL), mean ± SD	107 ± 36	98 ± 34	120 ± 36	<.001
Triglycerides (mg/dL), median (IQR)	135 (95–185)	137 (96–191)	130 (94–179)	.304
Hemoglobin (g/dL), mean ± SD	13.0 ± 2.0	13.4 ± 1.9	12.0 ± 1.4	<.001
Albumin (g/dL), mean ± SD	4.16 ± 0.52	4.17 ± 0.54	4.15 ± 0.49	.697
hs-CRP (mg/dL), median (IQR)	2.4 (1.0–5.5)	2.2 (1.0–4.7)	2.8 (1.2–6.4)	.049
Phosphate (mg/dL), mean ± SD	3.7 ± 0.8	3.6 ± 0.8	3.9 ± 0.8	<.001

SD: standard deviation; ACE: angiotensin-converting enzyme; BP: blood pressure; LDL: low-density lipoprotein; hs-CRP: high sensitivity-CRP.

## RESULTS

The study population included 759 patients with stages 2–5 CKD. Four hundred and fifty-five patients were males (60%) and the remaining 304 patients were females (40%). The proportion of smokers was about 4 times higher in males (71.4%) than in females (17.4%).

Males and females also differed for the prevalence of diabetes (38.5% versus 29.6%) and for the frequency of patients with background CV comorbidities (35.6% versus 19.7%, *P* < .001). Waist circumference (100.9 ± 12.4 cm versus 96 ± 14.1 cm), eGFR (37.5 ± 13.4 versus 33 ± 12.7 mL/min/1.73 m^2^), 24-h urinary protein excretion (median 0.7, IQR 0.2–1.6 g/24 h versus median 0.5, IQR 0.2–1.2 g/24 h) and haemoglobin (13.4 ± 1.9 versus 12.0 ± 1.4 g/dL) were higher in males than in females. On the contrary, serum phosphate (3.6 ± 0.75 versus 3.9 ± 0.75 mg/dL), high sensitivity-CRP (median 2.2, IQR 1–4.7 mg/dL versus median 2.8, IQR 1.2–6.4 mg/dL) and total cholesterol (178.3 ± 42.1 versus 198.8 ± 45.6 mg/dL) were lower in males than in females (Table 1). Moreover, body mass index (BMI) was significantly higher in females than in male CKD patients (28.7 ± 5.3 vs 27.8 ± 4.2 kg/m^2^, respectively, *P* = .022). No differences were observed for the remaining risk factors listed in Table 1.

### Survival analysis

**Figure 1:**
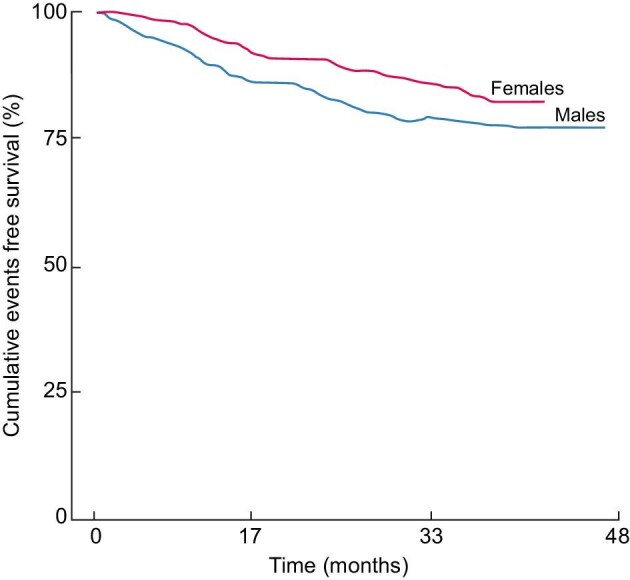
Kaplan–Meier analysis of fatal and non-fatal CV events according to sex. See ‘Results’ section for more detail.

**Figure 2:**
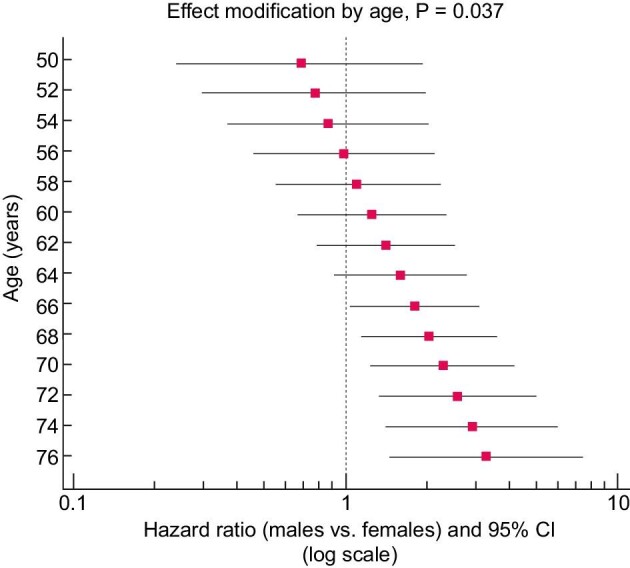
Effect modification analysis by age on the association between sex (males versus females) and fatal and nonfatal CV events. Data are HR and 95% CI. The *P*-value for effect modification was calculated by the linear combination method. See ‘Results’ section for more detail.

Across the follow-up period (median 36 months, range 0.3–48 months), 42 patients died and 118 patients had fatal and non-fatal CV events. On univariate Cox regression analyses, sex failed to be associated with all-cause mortality [HR (males versus females) 0.65, 95% CI 0.34–1.25, *P* = .20] whereas it was strongly related to the incidence rate of fatal and non-fatal CV events [HR (males versus females) 1.75, 95% CI 1.18–2.60, *P* = .006] (see Fig. 1). Of note, data adjustment for all potential confounders (see Table 2a and the ‘Statistical analysis’ section) did not materially affect the strength of the relationship between sex and the incidence rate of fatal and non-fatal CV outcomes which remained statistically significant [HR (males versus females) 1.78, 95% CI 1.03–3.09, *P* = .039]. Remarkably, a significant effect modification by age (*P* = .037) was found in the relationship between sex and the incidence rate of fatal and non-fatal CV outcomes (see Table 2b) so that the HR of CV events of males versus females increased in close parallel with age (Fig. 2). In a sensitivity analysis, forcing the allocation arm (active versus control) into the two multiple Cox models did not affect the relationship between sex and fatal and non-fatal CV events [HR (males versus females) 1.77, 95% CI 1.02–3.07, *P* = .042] as well as the significance of the effect modification by age on the relationship between sex and CV events (*P* = .037) (see Fig. 2).

**Table 2:** Multiple Cox regression models of fatal and non-fatal CV events.

Variables (units of increase)	HR	95% CI	*P*-value
**(a) Cox model of the main effect of sex**			
Sex (males versus females)	1.78	1.03–3.09	.039
Age (1 year)	1.07	1.03–1.09	<.001
Smoking (0 = no; 1 = yes)	1.33	0.83–2.13	.231
Diabetes (0 = no; 1 = yes)	1.17	0.77–1.76	.466
Previous CV events (0 = no; 1 = yes)	2.63	1.78–3.89	<.001
Waist circumference (1 cm)	1.01	0.99–1.02	.385
eGFR_MDRD186_ (1 mL/min/1.73 m^2^)	1.00	0.99–1.02	.827
Proteinuria (1 g/24 h)	1.08	0.97–1.21	.161
Cholesterol (1 mg/dL)	0.99	0.99–1.00	.819
LDL cholesterol (1 mg/dL)	1.00	0.99–1.01	.115
Haemoglobin (1 g/dL)	0.83	0.74–0.94	.003
hs-CRP (1 mg/dL)	0.99	0.97–1.01	.485
Phosphate (1 mg/dL )	1.18	0.93–1.51	.182
**(b) Cox model based on the effect modification by age on the sex**			
** outcomes link**			
Interaction term (age * sex)	1.06	1.00–1.12	.037
Sex (males versus females)	0.33	0.00–1.44	.077
Age (1 year)	1.02	0.98–1.07	.282
Other covariates			
Smoking (0 = no; 1 = yes)	1.26	0.78–2.01	0.342
Diabetes (0 = no; 1 = yes)	1.15	0.76–1.75	.497
Previous CV events (0 = no; 1 = yes)	2.73	1.84–4.05	<.001
Waist circumference (1 cm)	1.01	0.99–1.02	.306
eGFR_MDRD186_ (1 mL/min/1.73 m^2^)	1.00	0.99–1.02	.804
Proteinuria (1 g/24 h)	1.09	0.97–1.21	.150
Cholesterol (1 mg/dL)	1.00	0.99–1.01	.819
LDL cholesterol (1 mg/dL)	1.00	0.99–1.01	.130
Haemoglobin (1 g/dL)	0.83	0.74–0.94	.004
hs-CRP (1 mg/dL)	0.99	0.97–1.01	.521
Phosphate (1 mg/dL )	1.22	0.95–1.56	.118

LDL: low-density lipoprotein; hs-CRP: high sensitivity-CRP.

## DISCUSSION

Despite some evidence of sex-specific differences in epidemiology and outcomes in all stages of CKD, so far, scarce attention has been dedicated to many important complications in CKD patients such as CV complications, and particularly to CV outcomes related to the sex issue in this high-risk population. In this study, we aimed to address that knowledge gap by evaluating data on sex differences in CV outcomes in a southern Italian middle-aged CKD cohort. The most important findings in this southern cohort of CKD patients stage 2–5 were that males had a higher CV risk compared with women despite the fact that female patients had a relatively worse eGFR than males. Moreover, males differed from females because they had a higher prevalence of diabetes and background CV comorbidities than females. As far as modifiable risk factors are concerned among male CKD patients, the proportion of smokers was about 4 times higher in males than in females and this finding could signify that female patients were more compliant with medical recommendations or could just reflect what happens in the general population. In the whole CKD cohort from the south of Italy, a high prevalence of other modifiable risk factors was documented, such as overweight and obesity assessed by either BMI or waist circumference which is considered a better tool for the diagnosis of obesity in CKD patients [10]. The two groups did not differ for systolic blood pressure which was well within the target indicated by Current Guide-Lines for CKD patients (Table 1). Again, it is noteworthy that in this CKD cohort, the percentage of smoking and CV comorbidities was significantly higher in men than in women while they were comparable at the beginning of the observation for age, blood pressure and antihypertensive treatment. Another important note is that in the majority of studies, women are grossly underrepresented [11] while in our study the number of female participants was 40% of the whole population which is satisfactory for the analysis. In recent years, sex-specific issues and how they influence health have increasingly attracted public attention, as well as interest from those working with healthcare and prevention. We have learned that this topic is of great relevance not only in research but also in day-to-day medical practice. A clear example lies in the higher incidence of CV disease in men than in women of similar age, and the menopause-associated increase in CV disease in women. Globally, females are ∼30% more likely to have pre-dialysis chronic kidney disease (CKD) than males for reasons that are not fully understood. CKD is associated with numerous adverse health outcomes which makes understanding and working to eradicate sex-based disparities in CKD prevalence essential. This study maps both what is known, and what is unknown, about the way sex impacts the epidemiology and risk factors for CKD including age, diabetes, hypertension, obesity, smoking, cerebrovascular disease and CV events.

Although there is some evidence of sex-specific differences in epidemiology, diagnosis, natural history and outcomes in all stages of CKD, the data on sex differences in this special population are limited. As in both the general population and the Chronic Renal Insufficiency Cohort (CRIC) study [12], women tended to have more favourable CV risk profiles than men, including a lower prevalence of diabetes and a much lower prevalence of current or past smoking.

Another interesting finding which emerged in our study was the effect modification by age on the relationship between sex and fatal and non-fatal events. This interaction indicates that the magnitude of the HR of males versus females for CV events increases in close parallel with age. In particular, the excess risk of CV events in males versus females becomes significant when the age was higher than 65 years whereas no such difference was found when the age is below 65 years.

In our study, haemoglobin levels emerged as the third factor in rank order (see Table 2a) predicting the incidence rate of CV events in the study population. This finding is in keeping with other epidemiological and observational data and coherently indicates that low haemoglobin is an important determinant of adverse clinical outcomes in patients with CKD.

The finding that men with CKD have more CVD events than women is also consistent with previous research. However, it is important to note that the reasons for this sex difference are not fully understood and may be multifactorial. It is possible that differences in the pathophysiology of CVD in men and women may play a role, as well as differences in the prevalence and impact of non-traditional risk factors, such as psychosocial stress and hormonal factors.

Overall, the annual mortality rate observed in our study was about 2%, a figure two times higher than that observed in the Italian general population (annual mortality rate = 1%).

Overall, these results highlight the importance of considering sex differences in the assessment and management of CVD risk in CKD patients. Further research is needed to better understand the underlying mechanisms of these sex differences and to develop targeted interventions to reduce CVD risk in both men and women with CKD.

In conclusion, women with stage 2–5 CKD have a lower risk of CV events than men, partly due to their more favourable CV risk profile. Further studies and clinical trials should address the delicate issue of sex differences and CV outcomes in CKD patients not on dialysis.

## Data Availability

The data underlying this article will be shared on reasonable request to the corresponding author.

## References

[1] Swartling O , Yang Y, Clase CM et al. Sex differences in the recognition, monitoring, and management of CKD in health care: an observational cohort study. J Am Soc Nephrol 2022;33:1903–14. 10.1681/ASN.2022030373. 35906075 PMC9528319

[2] Jung CY , Heo GY, Park JT et al. Sex disparities and adverse cardiovascular and kidney outcomes in patients with chronic kidney disease: results from the KNOW-CKD. Clin Res Cardiol 2021;110:1116–27. 10.1007/s00392-021-01872-5. 34003323

[3] Maas AH , Appelman YE. Gender differences in coronary heart disease. Neth Heart J 2010;18:598–603. 10.1007/s12471-010-0841-y. 21301622 PMC3018605

[4] Nitsch D , Grams M, Sang Y et al.; Chronic Kidney Disease Prognosis Consortium. Associations of estimated glomerular filtration rate and albuminuria with mortality and renal failure by sex: a meta-analysis. BMJ 2013;346:f324. 10.1136/bmj.f324. 23360717 PMC3558410

[5] Padro T , Manfrini O, Bugiardini R et al. ESC Working Group on Coronary Pathophysiology and Microcirculation position paper on ‘coronary microvascular dysfunction in cardiovascular disease’. Cardiovasc Res 2020;116:741–55. 10.1093/cvr/cvaa003. 32034397 PMC7825482

[6] Champney KP , Frederick PD, Bueno H et al. The joint contribution of sex, age and type of myocardial infarction on hospital mortality following acute myocardial infarction. Heart 2009;95:895–9. 10.1136/hrt.2008.155804. 19147625 PMC3065924

[7] Neugarten J , Acharya A, Silbiger SR. Effect of sex on the progression of nondiabetic renal disease: a meta-analysis. J Am Soc Nephrol 2000;11:319–29. 10.1681/ASN.V112319. 10665939

[8] Weldegiorgis M , Woodward M. The impact of hypertension on chronic kidney disease and end-stage renal disease is greater in men than women: a systematic review and meta-analysis. BMC Nephrol 2020;21:506. 10.1186/s12882-020-02151-7. 33238919 PMC7687699

[9] Zoccali C , Leonardis D, Enia G et al. The MAURO study: multiple intervention and audit in renal diseases to optimize care. J Nephrol 2008;21:20–2. 18264932

[10] Sanches FM , Avesani CM, Kamimura MA et al. Waist circumference and visceral fat in CKD: a cross-sectional study. Am J Kidney Dis 2008;52:66–73. 10.1053/j.ajkd.2008.02.004. 18440683

[11] Bucholz EM , Krumholz HM. Women in clinical research: what we need for progress. Circ Cardiovasc Qual Outcomes 2015;8:S1–3. 10.1161/CIRCOUTCOMES.115.001756. 25714827 PMC4415379

[12] Toth-Manikowski SM , Yang W, Appel L et al.; Chronic Renal Insufficiency Cohort (CRIC) Study Investigators. Sex differences in cardiovascular outcomes in CKD: findings from the CRIC Study. Am J Kidney Dis 2021;78:200–9.e1. 10.1053/j.ajkd.2021.01.020. 33857532 PMC8316276

